# Risk of Late-Onset Depression in Long-Term Survivors of Breast, Prostate, and Colorectal Cancer

**DOI:** 10.1001/jamanetworkopen.2025.44812

**Published:** 2025-11-26

**Authors:** Melissa Taylor, Sarah J. Westvold, Jessica B. Long, Terry Hyslop, Andrea Silber, Rebecca Forman, Faiza Yasin, Tendai Kwaramba, Shi-Yi Wang, Michael S. Leapman, Michael Cecchini, Ira Leeds, Lisa Spees, Stephanie B. Wheeler, Cary P. Gross, Kevin Oeffinger, Michaela A. Dinan

**Affiliations:** 1 Department of Internal Medicine, Yale University School of Medicine, New Haven, Connecticut; 2 Yale Cancer Outcomes, Public Policy, and Effectiveness Research (COPPER) Center, New Haven, Connecticut; 3 Sidney Kimmel Cancer Center, Thomas Jefferson University, Philadelphia, Pennsylvania; 4 Department of Chronic Disease Epidemiology, Yale School of Public Health, New Haven, Connecticut; 5 Department of Urology, Yale University School of Medicine, New Haven, Connecticut; 6 Department of Surgery, Yale University School of Medicine, New Haven, Connecticut; 7 Lineberger Comprehensive Cancer Center, Chapel Hill, North Carolina; 8 Division of Pharmaceutical Outcomes and Policy, Eshelman School of Pharmacy, Lineberger Comprehensive Cancer Center, Chapel Hill, North Carolina; 9 Department of Health Policy and Management, Gillings School of Global Public Health, University of North Carolina at Chapel Hill, Chapel Hill; 10 Department of Medicine, Duke University, Durham, North Carolina; 11 Duke Cancer Institute, Duke University, Durham, North Carolina

## Abstract

**Importance:**

The prevalence of depression is higher in cancer survivors than in the general population. As the long-term cancer survivor population increases, it is increasingly important to understand risk factors for late-onset depression.

**Objective:**

To identify risk factors associated with late-onset depression in long-term (5-year) survivors of breast, prostate, or colorectal cancer.

**Design, Setting, and Participants:**

This retrospective cohort study used 2022 linkage of Surveillance, Epidemiology, and End Results (SEER) and Medicare data to identify 5-year survivors of breast, prostate, or colorectal cancer 66 years or older who were enrolled in fee-for-service Medicare and had no previous depression diagnosis. Long-term survivors were diagnosed with cancer between January 1, 2007, and December 31, 2012, and followed up between January 1, 2008, and December 31, 2020. Data analysis was performed from August 2024 to July 2025.

**Exposures:**

Sociodemographic and cancer-specific characteristics, treatment within 1 year after diagnosis, comorbidities within 1 year before cancer diagnosis, and previous diagnosis of anxiety.

**Main Outcomes and Measures:**

The primary outcome was late-onset depression 5 to 10 years after cancer diagnosis, identified using a validated claims-based algorithm. Analyses were conducted separately for each survivor cohort. Fine-Gray subdistribution hazard regression, accounting for the competing risk of mortality, was used to identify factors associated with late-onset depression.

**Results:**

A total of 53 769 survivors were identified, including 13 265 breast, 26 979 prostate, and 13 525 colorectal cancer survivors (mean [SD] age, 74.1 [5.8] years; 31 279 [61.9%] male; 2375 [4.4%] Asian or Pacific Islander, 2691 [5.0%] Hispanic, 3906 [7.3%] non-Hispanic Black, 43 986 [81.8%] non-Hispanic White, and 811 [1.5%] other or unknown) were identified. The 5-year risk of late-onset depression was highest in breast cancer survivors (13.3% [1768 of 13 265]), compared with prostate (8.7% [2360 of 26 979]) and colorectal (11.8% [1591 of 13 525]) cancer survivors. Older age was associated with greater hazard of depression among some categories (eg, prostate cancer survivors aged ≥90 years vs 71-74 years: HR, 1.57; 95% CI, 1.10-2.24) but not among others (eg, colorectal cancer survivors aged ≥90 years vs 71-74 years: HR, 1.02; 95% CI, 0.84-1.24). Variables that were consistently associated with greater hazard of depression included Medicare-Medicaid dual eligibility (eg, dual eligible breast cancer survivors vs non-dual eligible: HR, 1.38; 95% CI, 1.22-1.57), anxiety (eg, prostate cancer survivors: HR, 2.82; 95% CI, 2.47-3.22), and comorbidity burden (eg, breast cancer survivors: HR, 1.33; 95% CI, 1.12-1.57). In prostate cancer survivors, receipt of radiotherapy with or without androgen deprivation therapy was also associated with higher risk (HR, 1.22; 95% CI, 1.10-1.36). The risk of depression among survivors in the high-risk tertile was twice as high compared with the low-risk tertile.

**Conclusions and Relevance:**

In this cohort study of long-term cancer survivors, Medicare and Medicaid dual eligibility, higher comorbidity burden, and preexisting anxiety were independently associated with greater risk of late-onset depression. These findings suggest that these risk factors may be used to proactively inform survivorship care during the transition from cancer surveillance to preventive care, which could reduce the risk of inconsistent follow-up care for survivors that may drive socioeconomic and racial and ethnic disparities in depression screening and treatment.

## Key Points

**Question:**

What sociodemographic and clinical characteristics are associated with late-onset depression in long-term (5-year) survivors of breast, prostate, or colorectal cancer?

**Findings:**

This cohort study of 53 769 cancer survivors found that Medicare and Medicaid dual eligibility, a greater comorbidity burden at diagnosis, and a prior diagnosis of anxiety were associated with higher risk of depression in all survivor cohorts. The risk of depression among survivors in the high-risk tertile was twice as high compared with the low-risk tertile.

**Meaning:**

This study’s results suggest that these risk factors may be used to proactively inform screening and treatment of depression among cancer survivors.

## Teaser

This cohort study of survivors of breast, prostate, or colorectal cancer examines risk factors associated with late-onset depression.

## Introduction

Advances in cancer screening, diagnosis, and treatment have resulted in improved outcomes and a growing population of long-term (5-year) cancer survivors.^1^ However, the physical and psychological effects of cancer and long-term survivorship can pose a persistent risk of clinical depression or anxiety throughout survivorship.^2^ Physical effects, such as chronic pain and fatigue, changes in appetite and weight, and decline in cognitive function, are associated with clinical depression and can impact quality of life in older adults more severely compared with younger adults.^3,4,5^ Fear of cancer recurrence is common among cancer survivors and can lead to decreased quality of life, emotional distress, and issues with psychosocial adjustment.^6^ Extending beyond its physical and emotional impact, cancer can inflict changes in employment, social support networks, and insurance, contributing to financial distress, a common outcome among cancer survivors associated with reduced quality-of-life measures and greater risk of depression.^7,8^ Consequently, the prevalence of depression in people with cancer is estimated to be higher than in the general population.^9^

In 2014, the American Society of Clinical Oncology (ASCO) recognized that screening patients with cancer for psychological symptoms of depression and anxiety was critical to providing comprehensive care and adapted guidelines for screening and assessment of anxiety and depressive symptoms in patients with cancer.^10^ As survivors transition from medical oncology back to primary care, risk-stratified survivorship care models address potential care gaps by identifying high-risk individuals and directing them to appropriate pathways for managing late effects of cancer, such as depression.^11^ This study aimed to identify risk factors for late-onset depression in long-term (5-year) survivors of breast, prostate, or colorectal cancer.

## Methods

### Study Design and Data Source

The 2022 linkage of Surveillance, Epidemiology, and End Results (SEER) and Medicare data was used to identify long-term (5-year) survivors of breast, prostate, and colorectal cancer who were aged 66 years and older at cancer diagnosis. The SEER-Medicare data linkage, a collaborative effort between the National Cancer Institute and the Centers for Medicare & Medicaid Services (CMS), provides clinical and demographic information, cause of death, and Medicare claims for covered health services for persons with cancer.^12,13^ The Yale Institutional Review Board approved this study as exempt because it is secondary data analysis of information recorded such that participants cannot be reidentified; therefore, no informed consent was required. This study followed the Strengthening the Reporting of Observational Studies in Epidemiology (STROBE) reporting checklist.^14^

### Study Sample

Survivors were diagnosed between January 1, 2007, and December 31, 2012, and received definitive treatment within 1 year of cancer diagnosis, defined as surgery in the breast and colorectal cohorts and surgery or radiotherapy in the prostate cohort. Prostate cancer cases managed with active surveillance alone (no surgery or radiotherapy) were excluded. Survivors were required to maintain enrollment in Medicare Parts A and B fee for service from 1 year before diagnosis to year 5, after which they were censored early if they lost enrollment.

### Study Outcomes

Depression was measured from 1 year before through 10 years after cancer diagnosis and identified by a diagnosis code (*International Classification of Diseases, Ninth Revision [ICD-9] *or* International Statistical Classification of Diseases and Related Health Problems, Tenth Revision [ICD-10]*) for depression in any position on at least 1 inpatient or 2 outpatient claims 30 to 365 days apart. The CMS developed the depression chronic condition variable from validated algorithms.^15^ The algorithm for depressive disorder requiring 2 instances of diagnosis is estimated to have a positive predictive value of 0.88.^16^ *ICD-9* and *ICD-10* diagnosis codes for depression are listed in eTable 1 in Supplement 1.

Depression onset was identified by the first claim with a diagnosis code for depression. The period from 1 year before through 5 years after cancer diagnosis was treated as a washout period to accurately distinguish late-onset from prevalent depression.^17^ Patients with depression during the washout period were excluded. The follow-up period for late-onset depression began 5 years after diagnosis and extended up to year 10. We restricted the study to patients diagnosed with cancer after 2007, allowing all patients to be observed before and after the ASCO guideline change in 2014.

Deaths recorded as suicide or self-inflicted injury were counted as depression onset if they occurred before a depression diagnosis in claims data. Due to the small number of survivors with this cause of death (<11), these figures are not reported in compliance with SEER privacy standards.

### Independent Variables

Demographic variables included age at 5 years after cancer diagnosis, sex (colorectal cancer only), race and ethnicity (collected as a proxy for systemic racism), diagnosis year, zip code–level poverty, location of residency (metropolitan vs rural), and Medicare and Medicaid dual eligibility as a proxy for socioeconomic status. Disease characteristics at diagnosis, collected by SEER, included American Joint Committee on Cancer staging and grade (Gleason grade group in prostate cancer). Nodal status and hormone receptor status (estrogen and progesterone) were included for breast cancer, and prostate-specific antigen was included for prostate cancer.

Treatment was measured within 1 year of diagnosis and included chemotherapy and radiotherapy for breast and colorectal cancers. Receipt and duration of adjuvant hormone therapy were assessed for breast cancer using Medicare Part D claims from diagnosis up to year 5. Prostate cancer treatment was categorized as surgery, radiotherapy, radiotherapy with androgen deprivation therapy (ADT) for less than 6 months, and radiotherapy with ADT for more than 6 months. Late treatment was defined as receipt of chemotherapy, radiotherapy, or ADT after year 4 and before the start of follow-up at year 5. Ongoing ostomy in years 4 to 5 after diagnosis was defined based on the presence of claims for ostomy supplies in the Durable Medical Equipment files.

*ICD-9*, *ICD-10*, Healthcare Common Procedure Coding System, and *Current Procedural Terminology* codes were used to identify site-directed surgery and treatment variables (eTable 1 in Supplement 1). Comorbidities were assessed within 1 year of a precancer diagnosis using the Elixhauser Comorbidity Index^18^ and categorized as 0, 1 to 2, and 3 or more preexisting comorbidities. An anxiety diagnosis, measured with the same algorithm (2 outpatient or single inpatient diagnoses) used to define depression, occurring in the year before cancer diagnosis or between diagnosis and year 5, was included as a covariate.

### Statistical Analysis

Analyses were conducted separately for each survivor cohort. The least absolute shrinkage and selection operator (LASSO) with 10-fold cross validation was used as a regularization method to identify factors associated with late-onset depression and reduce model complexity. Data were split into training (70%) and validation (30%) groups with stratified random sampling to maintain even distribution of the outcome. We selected the model with minimum cross-validated error. Time-dependent receiver operating characteristic curves were used to assess the accuracy and generalizability of the model in the training and validation data.

Variables with coefficients greater than 0.01 in the LASSO models were included in Fine-Gray subdistribution hazard models accounting for mortality as a competing risk.^19^ We tested for proportional subdistribution hazards by adding an interaction with time and visually by plotting cumulative incidence functions for each covariate. Survivors who died before the end of follow-up or lost coverage were censored early.

Variables that were significantly associated with a higher hazard of late-onset depression were used to develop associated risk factor rules. Points were assigned based on the magnitude of regression coefficients, scaled relative to the smallest significant positive coefficient, and rounded to the nearest integer.^20,21^ Survivors were stratified into low-, intermediate-, and high-risk tertiles based on risk score distribution. The 5- to 10-year cumulative incidence of depression was calculated by risk tertile, accounting for mortality as a competing risk.^22^

All statistical tests were 2-sided at an α = .05, and 95% CIs that did not include 1 were considered statistically significant. Analyses were conducted in R, version 4.3.1 (R Project for Statistical Computing). The R package grpreg was used for group LASSO regularization.^23^ Data analysis was performed from August 2024 to July 2025.

## Results

### Cohort Characteristics

The final cohort included 53 769 survivors: 10 907 (14.9%) were excluded due to missing information on disease or demographic variables, and 8470 (13.6%) were excluded due to a depression diagnosis before year 5. Of these, 13 265 were diagnosed with breast cancer, 26 979 with prostate cancer, and 13 525 with colorectal cancer (eFigure 1 in Supplement 1). The mean (SD) age was 74.1 (5.8) years; 31 279 (61.9%) male and 20 490 (38.1%) female; 2375 (4.4%) Asian or Pacific Islander, 2691 (5.0%) Hispanic, 3906 (7.3%) non-Hispanic Black, 43 986 (81.8%) non-Hispanic White, and 811 (1.5%) other (including American Indian, Alaskan Native, and any race or ethnicity not otherwise specified) or unknown; and 9777 (18.2%) lived in an area with more than 20% of residents living below the federal poverty line.

Colorectal cancer survivors were older, with 4238 (31.3%) aged 85 years or older at the start of follow-up compared with breast (2997 [22.5%]) and prostate (1890 [7.0%]) cancer survivors. Medicare and Medicaid dual eligibility was more common among breast (2698 [20.3%]) and colorectal (2148 [15.9%]) cancer survivors compared with prostate cancer survivors (1942 [7.2%]). Colorectal cancer survivors had higher comorbidity burden, with 1922 (14.2%) having 3 or more compared with 1064 breast (8.0%) and 1669 prostate (6.2%) cancer survivors. A prior anxiety diagnosis was more common in breast (1274 [9.6%]) and colorectal (1263 [9.3%]) than in prostate (1221 [4.5%]) cancer survivors (Table 1).

**Table 1.** Characteristics of Long-Term Survivors of Breast, Prostate, Colon, and Rectal Cancer

Characteristic	No. (%) of cancer survivors		
	Breast (n = 13 265)	Prostate (n = 26 979)	Colorectal (n = 13 525)
**Demographic characteristics**			
Age group, y (5 years after diagnosis)			
69-74	3508 (26.4)	9525 (35.3)	2569 (19)
75-79	3816 (28.8)	10055 (37.3)	3551 (26.3)
79-84	2944 (22.2)	5509 (20.4)	3167 (23.4)
85-89	1950 (14.7)	1642 (6.1)	2502 (18.5)
≥90	1047 (7.9)	248 (0.9)	1736 (12.8)
Race and ethnicity^a^			
Asian or Pacific Islander	658 (5)	996 (3.7)	721 (5.3)
Hispanic	631 (4.8)	1407 (5.2)	653 (4.8)
Non-Hispanic Black	729 (5.5)	2321 (8.6)	856 (6.3)
Non-Hispanic White	11 058 (83.4)	21 858 (81)	11 070 (81.8)
Other or unknown	189 (1.4)	397 (1.5)	225 (1.7)
Area-level poverty, % of residents living below federal poverty line			
0-<5	3174 (23.9)	6949 (25.8)	3017 (22.3)
5-<10	3727 (28.1)	7987 (29.6)	3727 (27.6)
10-<20	3894 (29.4)	7447 (27.6)	4070 (30.1)
20-100	2470 (18.6)	4596 (17)	2711 (20)
Medicare-Medicaid dual eligibility^b^	2698 (20.3)	1942 (7.2)	2148 (15.9)
Location (metropolitan vs rural)			
Metropolitan	10 923 (82.3)	22 654 (84)	11 115 (82.2)
Rural	2342 (17.7)	22 654 (84.0)	11 115 (82.2)
Marital status			
Married	6483 (48.9)	22 192 (82.3)	7902 (58.4)
Not married	782 (51.1)	4787 (17.7)	5623 (41.6)
Sex			
Male	0	26 979	6300 (46.6)
Female	13 265	NA	7225 (53.4)
**Disease characteristics**			
Stage			
I	8409 (63.4)	2135 (7.9)	4505 (33.3)
II	3986 (30)	22 060 (81.8)	5341 (39.5)
III	870 (6.6)	2784 (10.3)	3679 (27.2)
Grade (Gleason prognostic grade group in prostate cohort)^c^			
I	3859 (29.1)	8949 (33.2)	1291 (9.5)
II	6359 (47.9)	13 373 (49.6)	9916 (73.3)
III	3047 (23)	4657 (17.3)	2318 (17.1)
Positive nodal status	2917 (22)	NA	3677 (27.2)
Hormone receptor status			
ER positive	11 691 (88.1)	NA	NA
PR positive	10 172 (76.7)	NA	NA
Prostate-specific antigen, ng/mL			
<10	NA	21 094 (78.2)	NA
10-20	NA	4279 (15.9)	NA
>20	NA	1606 (6)	NA
Colorectal cancer primary site			
Colon	NA	NA	10 809 (79.9)
Rectal	NA	NA	2716 (20.1)
Treatment			
Chemotherapy	2823 (21.3)	NA	NA
Radiation therapy	8457 (63.8)	NA	NA
Late treatment^d^	238 (1.8)	1256 (4.7)	446 (3.3)
Ostomy at year 4	NA	NA	585 (4.3)
Prostate cancer treatment			
Surgery	NA	9311 (34.5)	NA
Radiotherapy	NA	9433 (35)	NA
Radiotherapy and ADT (<6 mo)	NA	2002 (7.4)	NA
Radiotherapy and ADT (≥6 mo)	NA	6233 (23.1)	NA
Breast hormone therapy (duration)			
None	2944 (22.2)	NA	NA
Initiated (≤9 mo)	1660 (12.5)	NA	NA
>9 mo	8661 (65.3)	NA	NA
Diagnosis year of 2010-2012	6363 (48.0)	15 059 (55.8)	7540 (55.7)
**Other clinical characteristics**			
No. of comorbidities present at diagnosis (Elixhauser Comorbidity Index)			
0	7096 (53.5)	15 756 (58.4)	5923 (43.8)
1-2	5105 (38.5)	9554 (35.4)	5680 (42)
≥3	1064 (8.0)	1669 (6.2)	1922 (14.2)
Anxiety (before or after cancer diagnosis)	1274 (9.6)	1221 (4.5)	1263 (9.3)

Abbreviations: ADT, androgen deprivation therapy; ER, estrogen receptor; NA, not applicable; PR, progesterone receptor.

SI conversion factor: To convert prostate-specific antigen to micrograms per liter, multiply by 1.

^a^ Race and ethnicity data were obtained through the Master Beneficiary Summary File maintained by the Centers for Medicare & Medicaid Services. The race and ethnicity category of other or unknown includes American Indian, Alaskan Native, and any race or ethnicity not otherwise specified.

^b^ Dual eligibility was included as a proxy for socioeconomic status. Patients who became Medicare and Medicaid dual eligible for at least 1 month between the year before cancer diagnosis through 5 years after were considered dual eligible.

^c^ Per the Gleason grading system, prognostic grade group I includes patients with a Gleason score of 6 or less, group II includes patients with a Gleason score of 3 + 4 = 7, and group III includes patients with a Gleason score of 4 + 3 = 7. Prognostic grade group greater than III is collapsed into group III due to infrequent number of cases and constitutes patients with a score of 8 or higher.

^d^ Late treatment is defined as a claim for systemic therapy in years 4 to 5 after cancer diagnosis (year before the start of follow-up). This therapy includes chemotherapy and radiotherapy in breast, colon, and rectal cancer and ADT in prostate cancer.

**Figure 1.**
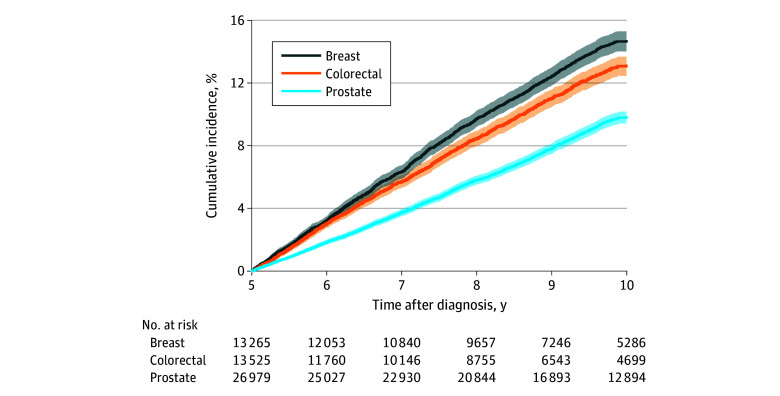
Cumulative Incidence of Late-Onset Depression in Long-Term Survivors of Breast, Prostate, and Colorectal Cancer at 5 and 10 Years Shaded areas indicate 95% CIs.

In the 5 to 10 years after cancer diagnosis, 5719 survivors (10.6%) were diagnosed with depression, 10 329 (19.2%) died of other causes, and 15 511 (28.8%) were censored early due to lost Medicare fee-for-servce enrollment. The 5-year risk of late-onset depression was highest in breast cancer survivors (13.3% [1768 of 13 265]) compared with prostate (8.7% [2360 of 26 979]) and colorectal (11.8% [1591 of 13 525]) cancer survivors (Figure 1).

### Model Performance

In the training data, the area under the receiver operating characteristic curve was 0.64 in the prostate and colorectal cohorts and 0.63 in the breast cohort. Performance in the validation data was consistent in all cohorts (eFigure 2A-C in Supplement 1).

### Factors Associated With Late-Onset Depression

#### Demographic Factors

In prostate cancer survivors, risk of late-onset depression increased with age: compared with survivors who were aged 71 to 74 years at 5 years after their cancer diagnosis, being 90 years or older was associated with 57% higher hazard of depression (hazard ratio [HR], 1.57; 95% CI, 1.10-1.24). In breast cancer survivors, being 75 to 84 years of age was associated with a higher hazard of depression (HR, 1.15; 95% CI, 1.00-1.32), but no other age groups were significantly associated with depression. In colorectal cancer survivors, being 85 to 89 years of age was associated with the highest hazard of depression (HR, 1.25; 95% CI, 1.06-1.49).

Compared with non-Hispanic White cancer survivors, non-Hispanic Black, Asian or Pacific Islander, and Hispanic survivors had lower hazards of late-onset depression across survivor cohorts. Medicare and Medicaid dual eligibility was associated with a higher hazard of depression in all cohorts, ranging from 25.0% higher in colorectal cancer survivors (HR, 1.25; 95% CI, 1.09-1.43) to 38.0% higher in breast cancer survivors (HR, 1.38; 95% CI, 1.22-1.57). In breast cancer survivors, residence in a high-poverty area was also associated with a higher hazard of depression (HR, 1.20; 95% CI, 1.02-1.40).

#### Disease and Treatment Factors

In all cohorts, disease characteristics were not associated with late-onset depression. In prostate cancer survivors, receipt of radiotherapy was associated with a 22.0% higher hazard of late-onset depression compared with surgery alone (HR, 1.22; 95% CI, 1.10-1.36). This association was 11% greater in survivors who also received ADT for more than 6 months (HR, 1.33; 95% CI, 1.18-1.50). In the colorectal cancer survivor cohorts, late treatment was associated with a 30.0% lower hazard of depression (HR, 0.70; 95% CI, 0.49-0.99). Conversely, in the prostate cancer survivor cohort late treatment (ADT) was associated with a 28.0% higher hazard of depression (HR, 1.28; 95% CI, 1.07-1.52).

#### Comorbidities

In all survivor cohorts, a greater comorbidity burden (≥3) was associated with a higher hazard of late-onset depression, ranging from 33.0% higher in the breast cancer cohort (HR, 1.33; 95% CI, 1.12-1.57) to 79.0% as high in the prostate cancer cohort (HR, 1.79; 95% CI, 1.55-2.08) compared with survivors with no comorbidities. A prior anxiety diagnosis was associated with the highest hazard of depression of all covariates. This association was greatest in prostate cancer survivors in whom their was a nearly 3 times higher hazard of depression (HR, 2.82; 95% CI, 2.47-3.22) (Table 2).

**Table 2.** Multivariable Fine-Gray Subdistribution Hazard Ratios for Late-Onset Depression in Older Long-Term Cancer Survivors With Competing Risk of Mortality

Characteristic	Hazard ratio (95% CI)		
	Breast cancer survivors	Prostate cancer survivors	Colorectal cancer survivors
**Demographic characteristics**			
Age 5 years after diagnosis, y			
71-74	1.00 [Reference]	1.00 [Reference]	1.00 [Reference]
75-79	0.98 (0.85-1.11)	1.07 (0.96-1.18)	1.18 (1.01-1.39)
79-84	1.15 (1.00-1.32)	1.20 (1.07-1.35)	1.18 (1.01-1.39)
85-89	1.16 (0.99-1.36)	1.22 (1.03-1.45)	1.25 (1.06-1.49)
≥90	1.11 (0.91-1.36)	1.57 (1.10-2.24)	1.02 (0.84-1.24)
Race^a^			
Asian or Pacific Islander	0.41 (0.30-0.55)	0.59 (0.45-0.78)	0.54 (0.40-0.72)
Hispanic	0.62 (0.48-0.80)	0.70 (0.56-0.87)	0.71 (0.54-0.92)
Non-Hispanic Black	0.65 (0.51-0.83)	0.62 (0.52-0.74)	0.76 (0.60-0.95)
Non-Hispanic White	1.00 [Reference]	1.00 [Reference]	1.00 [Reference]
Other or unknown	0.46 (0.27-0.80)	0.61 (0.40-0.93)	0.99 (0.67-1.46)
Area-level poverty, %			
0-<5	1.00 [Reference]	1.00 [Reference]	1.00 [Reference]
5-<10	1.07 (0.93-1.22)	0.97 (0.87-1.09)	Not selected
10-<20	1.09 (0.95-1.25)	1.05 (0.94-1.18)	Not selected
20-100	1.20 (1.02-1.40)	1.10 (0.97-1.26)	Not selected
Residence			
Nonmetropolitan or rural	1.00 [Reference]	1.00 [Reference]	1.00 [Reference]
Metropolitan	1.11 (0.98-1.27)	Not selected	Not selected
Marital status			
Married	1.00 [Reference]	1.00 [Reference]	1.00 [Reference]
Unmarried or single	1.05 (0.95-1.16)	1.11 (1.00-1.24)	Not selected
Medicare-Medicaid dual eligibility			
Not dual eligible	1.00 [Reference]	1.00 [Reference]	1.00 [Reference]
Dual eligible	1.38 (1.22-1.57)	1.38 (1.16-1.64)	1.25 (1.09-1.43)
Sex			
Male	NA	NA	1.00 [Reference]
Female	NA	NA	1.25 (1.13-1.39)
**Disease characteristics**			
Stage			
I	1.00 [Reference]	1.00 [Reference]	1.00 [Reference]
II	1.03 (0.90-1.17)	Not selected	Not selected
III	1.03 (0.81-1.31)	Not selected	Not selected
Grade			
I	1.00 [Reference]	1.00 [Reference]	1.00 [Reference]
II	0.94 (0.84-1.04)	1.04 (0.94-1.14)	1.05 (0.89-1.25)
III	0.87 (0.75-1.00)	1.01 (0.88-1.15)	1.09 (0.89-1.33)
Breast hormone receptor status			
ER positive (reference ER negative)	Not selected	NA	NA
PR positive (reference: PR negative)	Not selected	NA	NA
Nodal status			
Negative	1.00 [Reference]	NA	NA
Positive	1.14 (0.98-1.33)	NA	NA
Prostate-specific antigen, ng/mL			
<10	NA	1.00 [Reference]	NA
10-20	NA	Not selected	NA
>20	NA	Not selected	NA
Colorectal cancer primary site			
Colon	NA	NA	1.00 [Reference]
Rectal	NA	NA	0.95 (0.81-1.11)
Treatment			
Chemotherapy (reference: none)	0.93 (0.80-1.07)	NA	0.92 (0.81-1.05)
Radiotherapy (reference: none)	1.08 (0.97-1.19)	NA	0.90 (0.72-1.12)
Late treatment (reference: none)	1.09 (0.78-1.53)	1.28 (1.07-1.52)	0.70 (0.49-0.99)
Breast hormone therapy			
None	1.00 [Reference]	NA	NA
Initiated	1.05 (0.89-1.23)	NA	NA
>9 mo	0.93 (0.82-1.04)	NA	NA
Prostate cancer treatment			
Surgery	NA	1.00 [Reference]	NA
Radiotherapy	NA	1.22 (1.10-1.36)	NA
Radiotherapy and ADT <6 mo	NA	1.24 (1.05-1.46)	NA
Radiotherapy and ADT ≥6 mo	NA	1.33 (1.18-1.50)	NA
Diagnosis year			
2007-2009	1.00 [Reference]	1.00 [Reference]	1.00 [Reference]
2010-2012	1.10 (1.00-1.21)	1.19 (1.09-1.30)	Not selected
No. of comorbidities present at diagnosis (Elixhauser Comorbidity Index)			
0	1.00 [Reference]	1.00 [Reference]	1.00 [Reference]
1-2	1.25 (1.13-1.38)	1.32 (1.21-1.44)	1.26 (1.13-1.41)
≥3	1.33 (1.12-1.57)	1.79 (1.55-2.08)	1.54 (1.33-1.78)
Anxiety	1.99 (1.75-2.26)	2.82 (2.47-3.22)	1.91 (1.67-2.19)

Abbreviations: ADT, androgen deprivation therapy; ER, estrogen receptor; NA, not applicable; PR, progesterone receptor.

SI conversion factor: To convert prostate-specific antigen to micrograms per liter, multiply by 1.

^a^ Race and ethnicity data were obtained through the Master Beneficiary Summary File maintained by the Centers for Medicare & Medicaid Services. The race and ethnicity category of other or unknown includes American Indian, Alaskan Native, and any race or ethnicity not otherwise specified.

#### Risk Stratification

Associated risk factor rules for all cohorts included Medicare and Medicaid dual eligibility, number of comorbidities, and prior anxiety. Additional variables were added to the associated risk factor rules by cohort, including age, treatment, and marital status in prostate cancer survivors and high area-level poverty in breast cancer survivors. The maximum possible risk score was 15 in the breast cancer survivor cohort, 25 in the prostate cancer survivor cohort, and 10 in the colorectal cancer survivor cohort. The median (IQR) score was 2 (0-4) in breast cancer survivors, 4 (2-6) in prostate cancer survivors, and 2 (1-3) in colorectal cancer survivors (eTable 2 in Supplement 1).

**Figure 2.**
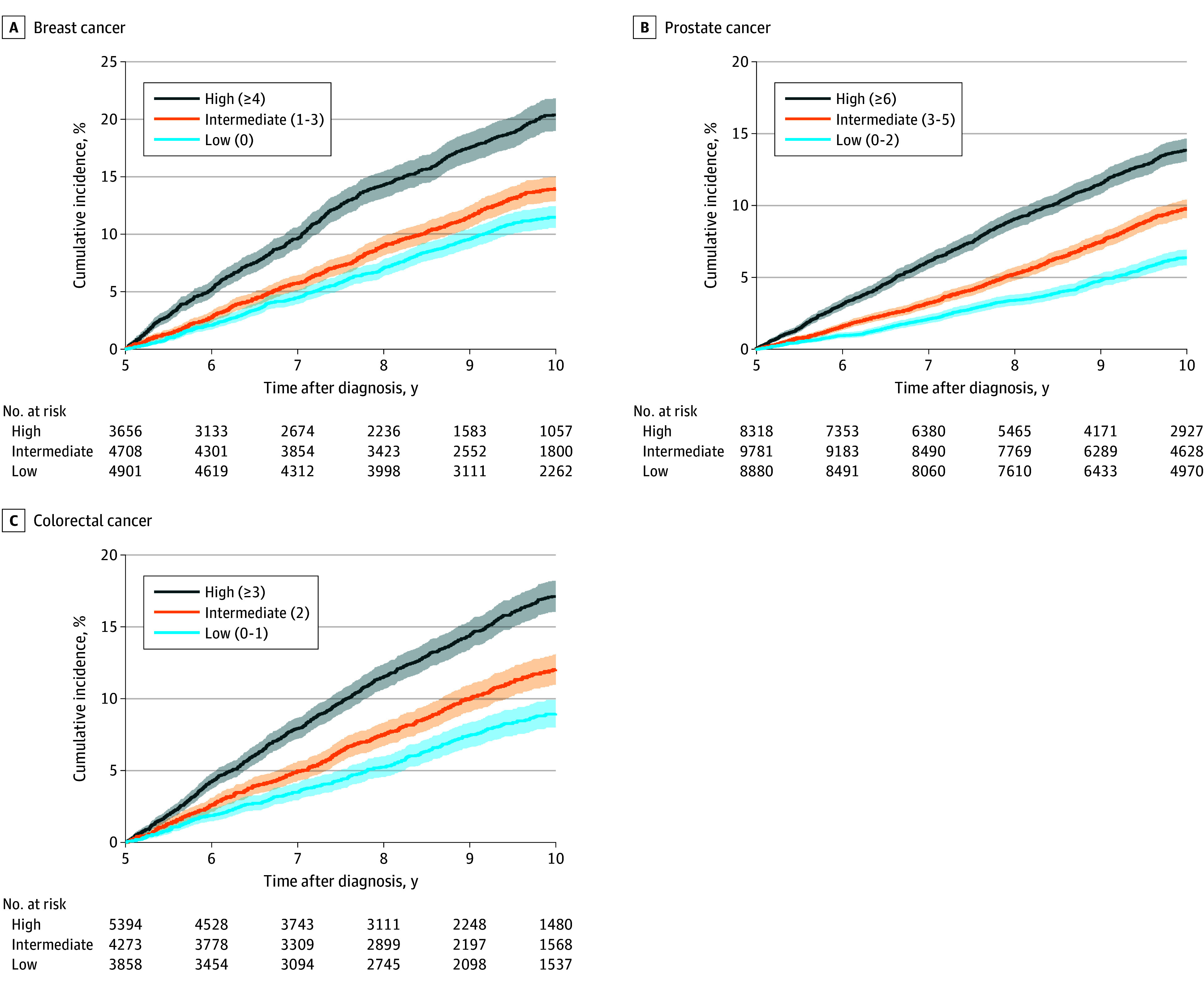
Cumulative Incidence of Late-Onset Depression by Risk Tertile at 5 and 10 Years Shaded areas indicate 95% CIs.

Across cohorts, the cumulative incidence of late-onset depression at year 10 after diagnosis in the high-risk tertile was twice as high as that in the low-risk tertile (Figure 2A-C). Breast cancer survivors in the high-risk tertile had the highest absolute risk of developing depression (661 of 3656 [20.4%]) during 5 years compared with prostate (1023 of 8318 [13.9%]) and colorectal (822 of 5394 [17.0%]) cancer survivors in the high-risk tertile.

Although Asian, Hispanic, and non-Hispanic Black cancer survivors had lower hazard of late-onset depression, a greater proportion were stratified to the high-risk tertile. In breast cancer survivors, 2505 of 11 058 non-Hispanic White survivors (22.7%) were identified as high risk compared with 729 of 490 non-Hispanic Black survivors (67.2%). This trend was similar for Hispanic survivors in the breast and colorectal cohorts (Table 3). Medicare and Medicaid dual eligibility was 3 to 6 times as high in non-Hispanic Black survivors compared with non-Hispanic White survivors (eTable 3 in Supplement 1).

**Table 3.** Cancer Survivor Characteristics by Late-Onset Depression Risk Tertile

Characteristic	No. (%) of cancer survivors								
	Breast			Prostate			Colorectal		
	Low risk (score, 0)	Intermediate risk (score, 1-3)	High risk (score, ≥4)	Low risk (score, 0-2)	Intermediate risk (score, 3-5)	High risk (score, ≥6)	Low risk (score, 0-1)	Intermediate risk (score, 2)	High risk (score, ≥3)
Total	4901 (36.9)	4708 (35.5)	3656 (27.6)	8880 (32.9)	9781 (36.2)	8318 (30.8)	3858 (28.5)	4273 (31.6)	5394 (39.9)
Race and ethnicity^a^									
Asian or Pacific Islander	171 (26.0)	230 (35.0)	257 (39.1)	227 (22.8)	350 (35.1)	419 (42.1)	152 (21.1)	210 (29.1)	359 (49.8)
Hispanic	90 (14.3)	190 (30.1)	351 (55.6)	271 (19.3)	458 (32.6)	678 (48.2)	147 (22.5)	179 (27.4)	327 (50.1)
Non-Hispanic Black	72 (9.9)	167 (22.9)	490 (67.2)	550 (23.7)	828 (35.7)	943 (40.6)	172 (20.1)	211 (24.6)	473 (55.3)
Non-Hispanic White	4506 (40.7)	4047 (36.6)	2505 (22.7)	7687 (35.2)	8008 (36.6)	6163 (28.2)	3323 (30.0)	3602 (32.5)	4145 (37.4)
Other or unknown	62 (32.8)	74 (39.2)	53 (28.0)	145 (36.5)	137 (34.5)	115 (29.0)	64 (28.4)	71 (31.6)	90 (40.0)
Area-level poverty, %									
0-<5	1592 (50.2)	1135 (35.8)	447 (14.1)	2429 (35.0)	2571 (37.0)	1949 (28.0)	901 (29.9)	1012 (33.5)	1104 (36.6)
5-<10	1740 (46.7)	1393 (37.4)	594 (15.9)	2827 (35.4)	2864 (35.9)	2296 (28.7)	1112 (29.8)	1202 (32.3)	1413 (37.9)
10-<20	1569 (40.3)	1475 (37.9)	850 (21.8)	2444 (32.8)	2709 (36.4)	2294 (30.8)	1211 (29.8)	1237 (30.4)	1622 (39.9)
≥20	0 (0)	705 (28.5)	1765 (71.5)	1180 (25.7)	1637 (35.6)	1779 (38.7)	634 (23.4)	822 (30.3)	1255 (46.3)
Marital status									
Unmarried or single	2929 (45.2)	2282 (35.2)	1272 (19.6)	8220 (37.0)	8138 (36.7)	5834 (26.3)	2828 (35.8)	2519 (31.9)	2555 (32.3)
Married	1972 (29.1)	2426 (35.8)	2384 (35.2)	660 (13.8)	1643 (34.3)	2484 (51.9)	1030 (18.3)	1754 (31.2)	2839 (50.5)
Stage									
I	3266 (38.8)	2980 (35.4)	2163 (25.7)	713 (33.4)	802 (37.6)	620 (29.0)	1286 (28.5)	1421 (31.5)	1798 (39.9)
II	1334 (33.5)	1427 (35.8)	1225 (30.7)	6798 (30.8)	8064 (36.6)	7198 (32.6)	1459 (27.3)	1701 (31.8)	2181 (40.8)
III	301 (34.6)	301 (34.6)	268 (30.8)	1369 (49.2)	915 (32.9)	500 (18.0)	1113 (30.3)	1151 (31.3)	1415 (38.5)
Grade									
I	1528 (39.6)	1356 (35.1)	975 (25.3)	3332 (37.2)	3293 (36.8)	2324 (26.0)	345 (26.7)	428 (33.2)	518 (40.1)
II	2331 (36.7)	2277 (35.8)	1751 (27.5)	4712 (35.2)	4790 (35.8)	3871 (28.9)	2926 (29.5)	3100 (31.3)	3890 (39.2)
III	1042 (34.2)	1075 (35.3)	930 (30.5)	836 (18.0)	1698 (36.5)	2123 (45.6)	587 (25.3)	745 (32.1)	986 (42.5)
Primary site									
Colon	NA	NA	NA	NA	NA	NA	2865 (26.5)	3418 (31.6)	4526 (41.9)
Rectal	NA	NA	NA	NA	NA	NA	993 (36.6)	855 (31.5)	868 (32.0)
ER status									
Negative	569 (36.1)	538 (34.2)	467 (29.7)	NA	NA	NA	NA	NA	NA
Positive	4332 (37.1)	4170 (35.7)	3189 (27.3)	NA	NA	NA	NA	NA	NA
PR status									
Negative	1140 (36.9)	1045 (33.8)	908 (29.4)	NA	NA	NA	NA	NA	NA
Positive	3761 (37.0)	3663 (36.0)	2748 (27.0)	NA	NA	NA	NA	NA	NA
Chemotherapy									
No	3841 (36.8)	3733 (35.7)	2868 (27.5)	NA	NA	NA	2735 (26.9)	3211 (31.6)	4229 (41.6)
Yes	1060 (37.5)	975 (34.5)	788 (27.9)	NA	NA	NA	1123 (33.5)	1062 (31.7)	1165 (34.8)
Radiotherapy									
No	1430 (29.7)	1749 (36.4)	1629 (33.9)	NA	NA	NA	3357 (27.5)	3868 (31.7)	4975 (40.8)
Yes	3471 (41.0)	2959 (35.0)	2027 (24.0)	NA	NA	NA	501 (37.8)	405 (30.6)	419 (31.6)
Breast hormone therapy									
None	1039 (35.3)	1070 (36.3)	835 (28.4)	NA	NA	NA	NA	NA	NA
Initiated ≤9 mo	601 (36.2)	585 (35.2)	474 (28.6)	NA	NA	NA	NA	NA	NA
Initiated >9 mo	3261 (37.7)	3053 (35.2)	2347 (27.1)	NA	NA	NA	NA	NA	NA
ADT duration, mo									
None	NA	NA	NA	7919 (44.4)	6243 (35.0)	3675 (20.6)	NA	NA	NA
<6	NA	NA	NA	703 (29.1)	928 (38.4)	788 (32.6)	NA	NA	NA
6-18	NA	NA	NA	209 (4.5)	1831 (39.4)	2611 (56.1)	NA	NA	NA
>18	NA	NA	NA	49 (2.4)	779 (37.6)	1244 (60.0)	NA	NA	NA
Colorectal cancer surgery type									
Right colectomy	NA	NA	NA	NA	NA	NA	1132 (24.4)	1471 (31.7)	2042 (44.0)
Left colectomy	NA	NA	NA	NA	NA	NA	1267 (27.5)	1444 (31.4)	1894 (41.1)
Total colectomy	NA	NA	NA	NA	NA	NA	54 (30.7)	52 (29.5)	70 (39.8)
Low anterior resection	NA	NA	NA	NA	NA	NA	994 (33.8)	950 (32.3)	1000 (34.0)
abdominoperineal resection	NA	NA	NA	NA	NA	NA	263 (38.1)	207 (30.0)	221 (32.0)
Local excision or other	NA	NA	NA	NA	NA	NA	148 (31.9)	149 (32.1)	167 (36.0)
Ostomy at year 4									
No	NA	NA	NA	NA	NA	NA	3651 (28.2)	4083 (31.6)	5206 (40.2)
Yes	NA	NA	NA	NA	NA	NA	207 (35.4)	190 (32.5)	188 (32.1)

Abbreviations: ADT, androgen deprivation therapy; ER, estrogen receptor; NA, not applicable; PR, progesterone receptor.

^a^ Race and ethnicity data were obtained through the Master Beneficiary Summary File maintained by the Centers for Medicare & Medicaid Services. The race and ethnicity category of other or unknown includes American Indian, Alaskan Native, and any race or ethnicity not otherwise specified.

## Discussion

In this study, we examined new-onset depression occurring 5 years after cancer diagnosis among older long-term cancer survivors. Factors associated with late-onset depression included age, dual Medicare-Medicaid eligibility, greater comorbidity burden, and a history of anxiety. Disease-related factors were not associated with late-onset depression. Treatment-related risk was noted in prostate cancer survivors, but no association with treatment was observed for breast or colorectal cancer survivors.

The 5- to 10-year risk of late-onset depression was 14.7% in breast cancer, 9.9% in prostate cancer, and 13.2% in colorectal cancer survivors. This finding is consistent with prior literature reporting long-term depression prevalence ranging from 10% to 24% across cancer types, higher than in older adults without cancer (approximately 5%).^9,20,21,22,23,24,25,26,27^ ASCO estimates that the prevalence of major depression in patients with cancer is 14.3% based on diagnostic criteria and 21.0% to 33.0% based on self-report of symptom measures.^9^

The association of dual Medicare-Medicaid eligibility with late-onset depression reflects known associations between low-income socioeconomic status and mental health burden. Dual-eligible patients often face fragmented care, lower health care plan reimbursement, and limited access to mental health services.^24,25^ Dual-eligible patients often have more complex care needs, leading to frequent interactions with the health care system and potentially greater opportunity for depression to be identified as a diagnosis.^26^ Although dual coverage could improve access to outpatient mental health services, the complexity of navigating 2 programs and lower reimbursement for psychosocial services often limit effective depression care.^27^ Additionally, financial hardship is a well-documented challenge among cancer survivors, often resulting from prolonged treatment-related costs, employment disruptions, and limited access to supportive services.^28^ Prior work demonstrates that older adults with cancer who experience financial hardship are twice as likely to report depressive symptoms, even after adjusting for sociodemographic and health factors.^29^ Longitudinal data suggest that the association is unidirectional in that financial distress is associated with subsequent depressive symptoms.^30^

Across all cohorts, non-Hispanic Black and Hispanic cancer survivors had lower hazards of late-onset depression compared with non-Hispanic White cancer survivors. Although this finding is consistent with prior evidence on lifetime prevalence, underdiagnosis and undertreatment in these groups remain a concern.^31,32^ Prior research shows that disparities in depression screening and treatment contribute to the underdiagnosis and undertreatment of depression in racial and ethnic minority groups and can result in more severe symptoms of depression at diagnosis.^33^ Patients with language barriers and older adults are particularly at risk of the underrecognition of depressive symptoms.^34,35^ Inequities in screening and treatment practices may partially explain why non-Hispanic Black and Hispanic race and ethnicity were associated with lower risk of depression, yet most non-Hispanic Black and Hispanic cancer survivors were stratified to high-risk tertiles. Dual eligibility and higher comorbidity burden were more common among non-Hispanic Black and Hispanic cancer survivors compared with non-Hispanic White cancer survivors, suggesting that socioeconomic disparities may contribute to depression risk in these groups.

In prostate cancer survivors, radiotherapy and ADT were associated with the highest risk of depression compared with prostate surgery alone. The finding that prolonged hormone therapy is associated with late-onset depression is consistent with a prior SEER-Medicare study that found that prostate cancer survivors receiving ADT had a 23% higher risk of depression compared with those not receiving ADT, with risk increasing with longer durations of therapy.^36^ Prospective studies of patient-reported outcomes show tradeoffs in quality-of-life metrics, such as bowel, urinary, and sexual function, but do not demonstrate a consistent disadvantage for radiotherapy alone compared with prostatectomy.^37,38^ Among men receiving treatment, radiotherapy is preferentially administered to older men with more comorbidities: in our cohort, men who received radiotherapy were a mean of 4 years older than those who received surgery. The association between radiotherapy alone (without ADT) and depression could be in part due to confounding by age and comorbidities, both of which were associated with the risk of depression in this cohort. Our study is limited to patients who received surgery or radiotherapy; therefore, we cannot assess whether these treatments carry a higher risk of depression compared with active surveillance.

Comorbidities were associated with late-onset depression, aligning with prior studies showing that depression risk increases with the number of physical comorbidities present at diagnosis.^39,40^ This finding is particularly relevant in older adults, in whom age and multimorbidities can compound to worsen quality of life and increase health care burden, contributing to psychosocial risk factors.^41,42^ However, there is limited research on how specific comorbidities and their long-term management affect depression risk beyond 5 years in cancer survivors 65 years and older, highlighting a need for further research in this area.

### Limitations

This study has some limitations. Our sample was predominately non-Hispanic White, which may limit the generalizability of findings to different racial and ethnic populations. The use of claims-based data to define depression could underestimate its true prevalence, particularly given known underdiagnosis and undertreatment among minority populations. Additionally, claims data may be incomplete or inconsistently coded and lack important clinical details, such as family history, substance use, social support, or history of trauma, which may influence depression risk.

## Conclusions

In this cohort study of long-term cancer survivors, socioeconomic vulnerability and multiple medical comorbidities were risk factors associated with late-onset depression 5 to 10 years after diagnosis. These data may be used to proactively inform survivorship care during the transition from cancer surveillance to preventive care, which could reduce the risk of inconsistent follow-up care for survivors that may drive socioeconomic and racial and ethnic disparities in depression screening and treatment. Further research is needed to explore how socioeconomic disadvantage intersects with race and ethnicity to drive disparities in late-onset depression and to inform more equitable, targeted interventions for high-risk populations.

## Notes

Supplement 1. **eFigure 1. **Sample Selection **eFigure 2. **LASSO Model Performance (AUC-ROC) in the Training (70%) and Validation (30%) Data at Year **eTable 1. **International Classification of Disease (ICD) -9 and -10, Healthcare Common Procedure Coding System (HCPCS), and Common Procedural Terminology (CPT) Codes Used to Identify Depression Outcome, Covariates, and Definitive Treatment in Medicare Claims **eTable 2. **Risk Score for Late Depression by Survivor Cohort **eTable 3. **Depression Risk Factors Stratified by Race and Ethnicity

Supplement 2.
Data Sharing Statement
