# Supplementary material for: Risk of Late-Onset Depression in Long-Term Survivors of Breast, Prostate, and Colorectal Cancer
Source: JAMA Netw Open. 2025 Nov 26;8(11):e2544812. doi: 10.1001/jamanetworkopen.2025.44812 (PMC12658655; doi:10.1001/jamanetworkopen.2025.44812)
Supplement: Supplement 1. — eFigure 1. Sample Selection eFigure 2. LASSO Model Performance (AUC-ROC) in the Training (70%) and Validation (30%) Data at Year eTable 1. International Classification of Disease (ICD) -9 and -10, Healthcare Common Procedure Coding System (HCPCS), and Common Procedural Terminology (CPT) Codes Used to Identify Depression Outcome, Covariates, and Definitive Treatment in Medicare Claims eTable 2. Risk Score for Late Depression by Survivor Cohort eTable 3. Depression Risk Factors Stratified by Race and Ethnicity [file jamanetwopen-e2544812-s001.pdf]

Supplemental Online Content

Taylor M, Westvold SJ, Long JB, et al. Risk of late-onset depression in long-term survivors of breast, cancer, and colorectal cancer. *JAMA Netw Open*. 2025;8(11):e2544812.
doi:10.1001/jamanetworkopen.2025.44812

eFigure 1. Sample Selection

eFigure 2a-c. LASSO Model Performance (AUC-ROC) in the Training (70%) and Validation (30%) Data at Year

eTable 1. International Classification of Disease (ICD) -9 and -10, Healthcare Common Procedure Coding System (HCPCS), and Common Procedural Terminology (CPT) Codes Used to Identify Depression Outcome, Covariates, and Definitive Treatment in Medicare Claims

eTable 2. Risk Score for Late Depression by Survivor Cohort

eTable 3. Depression Risk Factors Stratified by Race and Ethnicity

This supplemental material has been provided by the authors to give readers additional information about their work.

© 2025 Taylor M et al. *JAMA Network Open.*

eFigure 1. Sample Selection

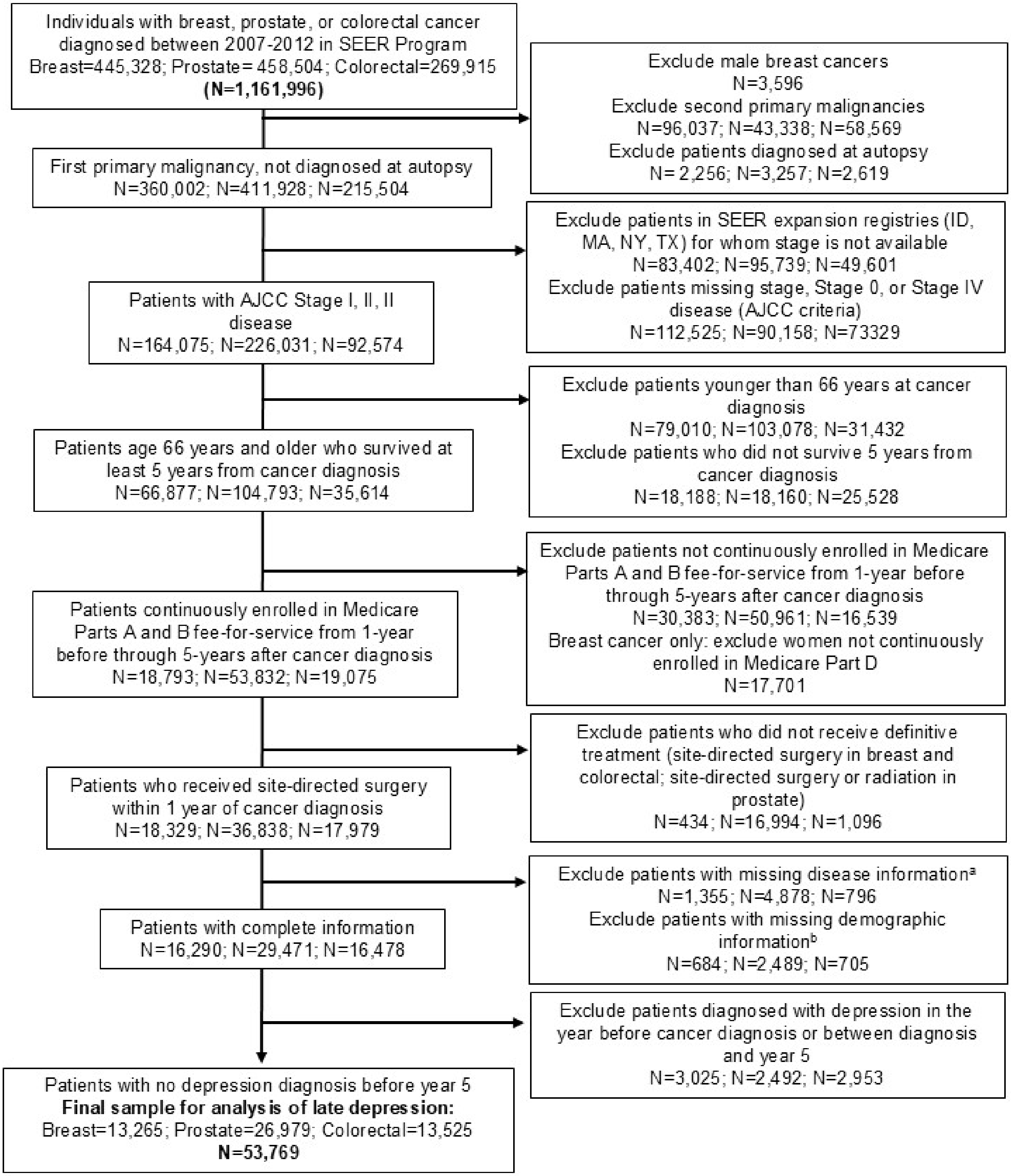

Footnotes

[a]Missing disease information includes grade, nodal status, estrogen receptor status, or progesterone receptor status for breast; Gleason grade or prostate specific antigen (PSA) for prostate; grade for colorectal cancer

[b]Missing demographic information includes marital status, location (metropolitan vs. rural), and area-level poverty for all cohorts

© 2025 Taylor M et al. *JAMA Network Open.*

eFigure 2A-C. LASSO Model Performance (AUC-ROC) in the training (70%) and validation (30%) data at year 10

a. Breast

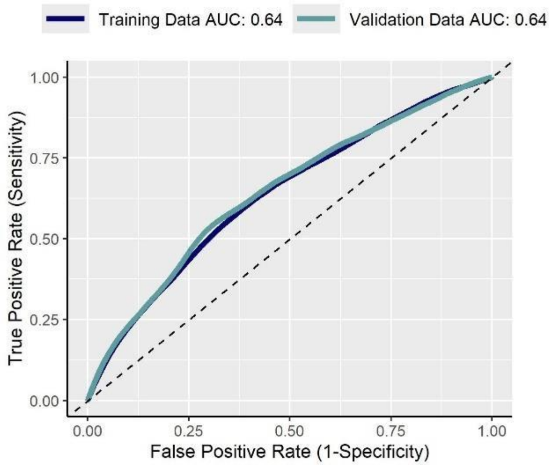

b. Prostate

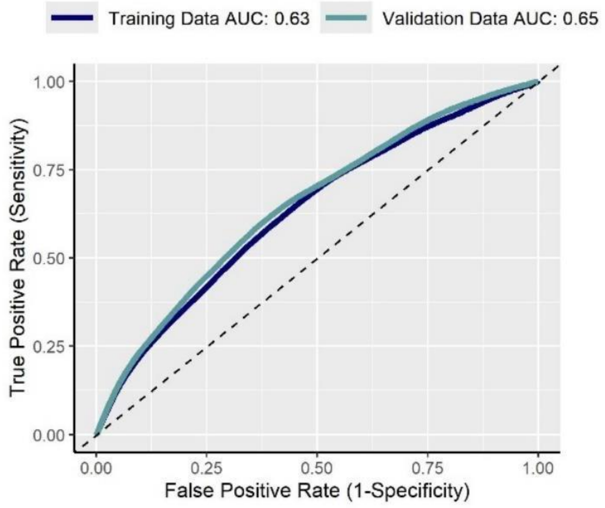

c. Colorectal

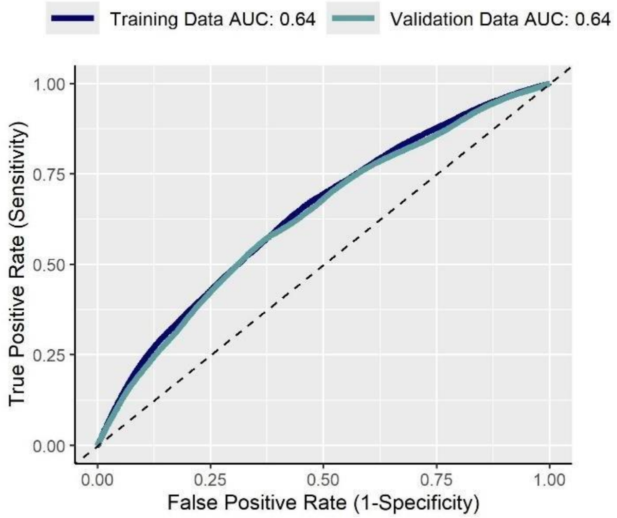

© 2025 Taylor M et al. *JAMA Network Open.*

eTable 1. International Classification of Disease (ICD) -9 and -10, Healthcare Common Procedure Coding System (HCPCS), and Common Procedural Terminology (CPT) codes used to identify depression outcome, covariates, and definitive treatment in Medicare Claims

**ICD-9 and ICD-10 codes used to identify depression and anxiety**	
**Depression[a]**	
ICD-9 Codes	311, 2980, 3004, 3091, 29620, 29621, 29622, 29623, 29624, 29625, 29626, 29630, 29631, 29632, 29633, 29634, 29635, 29636, 29651, 29652, 29653, 29654, 29655, 29656, 29660, 29661, 29662, 29663, 29664, 29665, 29666, 29689
ICD-10 Codes	F314, F315, F320, F321, F322, F323, F324, F325, F329, F330, F331, F332, F333, F338, F339, F341, F432, F433, F3130, F3131, F3132, F3160, F3161, F3162, F3163, F3164, F3175, F3176, F3177, F3178, F3240, F3241, F3340, F3341, F3342
**Anxiety[a]**	
ICD-9 Codes	3003, 3005, 3009, 3080, 3081, 3082, 3083, 3084, 3089, 3130, 3131, 3133, 29384, 30000, 30001, 30002, 30009, 30010, 30020, 30021, 30022, 30023, 30029, 30089, 30800, 30810, 30820, 30830, 30840, 30981, 31321, 31322, 31382, 31383
ICD-10 Codes	F4000, F4001, F4002, F4010, F4011, F40210, F40218, F40220, F40228, F40230, F40231, F40232, F40233, F40240, F40241, F40242, F40243, F40248, F40290, F40291, F40298, F4310, F4311, F4312, F064, F400, F401, F408, F409, F410, F411, F413, F418, F419, F42, F422, F423, F424, F428, F429, F430, F43, F431, F448, F488, F489, F938, F99, R452, R455, R456, R457

HCPCS and CPT codes used to identify treatment and surgery in Medicare Claims

Variable	HCPCS or CPT Codes	Generic drug name/Definition
Chemotherapy	HCPCS: J9151, J9150, J9000, J9002, Q2048, Q2049, Q2050, J9001, J9178, J9211, J9270, C9265, C9280, J0894, J8610, J8999, J9017, J9025, J9033, J9040, J9041, J9045, J9047, J9060, J9062, J9070, J9080, J9090, J9091, J9092, J9093, J9094, J9095, J9096, J9097, J9100, J9130, J9170, J9171, J9179, J9181, J9182, J9185, J9190, J9200, J9201, J9206, J9207, J9208, J9245, J9250, J9260, J9263, J9264, J9265, J9267, J9280, J9290, J9293, J9305, J9315, J9340, J9351, J9360, J9370, J9375, J9390, J9999	Daunorubicin, Doxorubicin, Epirubicin, Idarubicin, Valrubicin, Romidepsin, Eribulin mesylate, Decitabine, Methotrexate, Chemotherapy - non specific, Arsenic Trioxide, Azacitidine, Bendamustine Hcl, Bleomycin, Bortezomib, Carboplatin, Carfilzomib, Cisplatin, Cyclophosphamide, Cytarabine, Dacarbazine, Docetaxel, Etoposide, Fludarabine, Fluorouracil, Floxuridine, Gemcitabine, Irinotecan, ixabepilone, Ifosfamide, Melphalan, Oxaliplatin, Paclitaxel, Mitomycin, Mitoxantrone, Pemetrexed, Thiotepa, Topotecan, Vinblastine, Vincristine, Vinorelbine
Radiation	77402-77414, 77416	Radiation treatment delivery
	0073T, 77385, 77386, 77418	IMRT delivery
	77761-77763	Intracavitary radiation therapy
	77770-77772, 77781-77787, 77799, 0182T	Clinical brachyterapy radiation treatment, high dose rate electronic brachytherapy
	77776-77778	Interstitial radiation therapy
Breast hormone	therapy	S0170, S0156, J9395, J9393, J9394, J9226, J9225, J1675,

© 2025 Taylor M et al. *JAMA Network Open.*

J1950, J9217 - J9219, C9430, J1952, S0187, J3315		Anastrozole, Elacestrant, Exemestane, Fulvestrant, Goserelin, Histerelin, Letrozole, Leuprolide, Raloxifene, Tamoxifen, Toremifene, Triptorelin

© 2025 Taylor M et al. *JAMA Network Open.*

Androgen deprivation therapy	J9202, J3315, J9155, J1950, J9217, J9218	Goserelin implant,triptorelin injection, degarelix injection, leuprolide acetate injection
**Surgery**[b]		
Breast cancer	19140, 19300, 19180, 19182, 19120, 19125, 19126, 19160 , 19162, 19301, 19302, 19200, 19220, 19240, 19303 - 19307, 19110	Removal of breast tissue, excision of breast leison, removal of breast, partial mastectomy, complete mastectomy
Prostate cancer	55801, 55821, 55831, 55866, 55840, 55842, 55845, 55810 , 55812 , 55815 , 55837 , 54520, 54522, 54530, 54535, 54690	Excision procedures on the prostate, prostatectomy, radical prostatectomy, cryosurgical ablation of prostate, orchiectomy
Colon & rectal cancer	44140, 44141, 44143- 44146, 44147, 44150-44158, 44160,44202, 44204-44208, 44210-44212, 45110-45114, 45116,45119-45121, 45123, 45126, 45395, 45397, 45160, 45170-45172	Left colectomy, right colectomy, total colectomy, local tumor excision, lower anterior resection, abdominoperineal resection

Footnotes

[a]ICD-9 and ICD-10 codes used to identify depression and anxiety in Medicare claims data are from the validated algorithm made available by the Chronic Conditions Warehouse. We identified depression and anxiety as a diagnosis code on one inpatient or two outpatient claims 30 to 365 days apart. The date of depression onset was recorded as the date of the first claim with a depression diagnosis code.

[b]Surgery codes were used to identify site-directed surgery as definitive treatment which was required for inclusion in the final sample. In the prostate cohort only, radiation was considered definitive treatment.

© 2025 Taylor M et al. *JAMA Network Open.*

eTable 2. Risk Score for Late Depression by Survivor Cohort

	Breast	Prostate	Colorectal
**Age 5-years post-diagnosis**			
75-79	—	—	+1
79-84	—	+2	+1
85-89	—	+2	+1
90+	—	+4	—
**Area-level poverty**			
>20% of residents	+2	—	—
**Medicare and Medicaid dual eligibility**			
Dual-eligible	+3	+3	+1
**Sex**			
Female	—	—	+1
**Marital status**			
Unmarried/Single	—	+1	—
**Prostate Cancer Treatment**			
Radiation monotherapy or in combination with short term ADT (< 6 months)	—	+2	—
Radiation with ADT for ≥6 months	—	+3	—
**Late treatment**			
ADT 4-5 years post-diagnosis	—	+2	—
**Comorbidities present at diagnosis**			
1-2	+2	+3	+1
≥3	+3	+5	+3
**Anxiety** Pre-cancer or between cancer diagnosis and year 5	+7	+10	+4
**Risk score distribution**			
Median [IQR]	2 [0, 4]	4 [2, 6]	2 [1, 3]
Range (Min, Max)	(0, 15)	(0, 25)	(0, 10)
**Risk tertile score range[a]**			
Low risk	0	0-2	0-1
Intermediate risk	1-3	3-5	2
High risk	≥4	≥6	≥3

[a]Risk groups were separated based on tertiles using the closest integer thus the actual percent in each category varies from 33%.

© 2025 Taylor M et al. *JAMA Network Open.*

eTable 3. Depression risk factors stratified by race and ethnicity

	Non-Hispanic White	Non-Hispanic Black	Asian	Hispanic	Other/Unknown
**Breast Cancer** (N)	11058 (83.4)	729 (5.5)	658 (5)	631 (4.8)	189 (1.4)
Area-level poverty					
0-<5%	2824 (25.5)	34 (4.7)	187 (28.4)	76 (12)	53 (28)
5-<10%	3268 (29.6)	86 (11.8)	203 (30.9)	113 (17.9)	57 (30.2)
10-<20%	3275 (29.6)	197 (27)	169 (25.7)	209 (33.1)	44 (23.3)
20-100%	1691 (15.3)	412 (56.5)	99 (15)	233 (36.9)	35 (18.5)
Medicare/Medicaid Dual Eligibility					
Not dual eligible	9576 (86.6)	271 (37.2)	347 (52.7)	241 (38.2)	132 (69.8)
Dual eligible	1482 (13.4)	458 (62.8)	311 (47.3)	390 (61.8)	57 (30.2)
Comorbidities present at diagnosis					
None	6112 (55.3)	276 (37.9)	311 (47.3)	299 (47.4)	98 (51.9)
1-2	4127 (37.3)	338 (46.4)	292 (44.4)	272 (43.1)	76 (40.2)
≥3	819 (7.4)	115 (15.8)	55 (8.4)	60 (9.5)	15 (7.9)
**Prostate Cancer** (N)	21,858 (81)	2,321 (8.6)	996 (3.7)	1,407 (5.3)	397 (1.5)
Marital status					
Married	22192 (82.3)	18325 (83.8)	1581 (68.1)	861 (86.4)	1093 (77.7)
Unmarried/Single	4787 (17.7)	3533 (16.2)	740 (31.9)	135 (13.6)	314 (22.3)
Medicare/Medicaid Dual Eligibility					
Not dual eligible	25037 (92.8)	21214 (97.1)	1954 (84.2)	702 (70.5)	813 (57.8)
Dual eligible	1942 (7.2)	644 (2.9)	367 (15.8)	294 (29.5)	594 (42.2)
Prostate Cancer Treatment					
Surgery	9311 (34.5)	7732 (35.4)	550 (23.7)	363 (36.4)	500 (35.5)
Radiation	9433 (35)	7573 (34.6)	1034 (44.5)	283 (28.4)	443 (31.5)
Radiation + ADT < 6 months	2002 (7.4)	1634 (7.5)	172 (7.4)	73 (7.3)	98 (7)
Radiation + ADT >6 months	6233 (23.1)	4919 (22.5)	565 (24.3)	277 (27.8)	366 (26)
Comorbidities present at diagnosis					
None	15756 (58.4)	13097 (59.9)	1153 (49.7)	515 (51.7)	762 (54.2)
1-2	9554 (35.4)	7543 (34.5)	912 (39.3)	415 (41.7)	537 (38.2)
≥3	1669 (6.2)	1218 (5.6)	256 (11)	66 (6.6)	108 (7.7)
**Colorectal cancer** (N)	11070 (81.8)	856 (6.3)	721 (5.3)	653 (4.8)	225 (1.7)
Medicare/Medicaid Dual Eligibility					
Not dual eligible	9909 (89.5)	554 (64.7)	370 (51.3)	373 (57.1)	171 (76)
Dual eligible	1161 (10.5)	302 (35.3)	351 (48.7)	280 (42.9)	54 (24)
Sex					
Male	5167 (46.7)	342 (40)	344 (47.7)	335 (51.3)	112 (49.8)
Female	5903 (53.3)	514 (60)	377 (52.3)	318 (48.7)	113 (50.2)
Comorbidities present at diagnosis					
None	4962 (44.8)	297 (34.7)	320 (44.4)	245 (37.5)	99 (44)
1-2 Comorbidities	4589 (41.5)	396 (46.3)	301 (41.7)	297 (45.5)	97 (43.1)
≥3	1519 (13.7)	163 (19)	100 (13.9)	111 (17)	29 (12.9)

© 2025 Taylor M et al. *JAMA Network Open.*
