# Supplementary material for: Risk of Late-Onset Depression in Long-Term Survivors of Breast, Prostate, and Colorectal Cancer
Source: JAMA Netw Open. 2025 Nov 26;8(11):e2544812. doi: 10.1001/jamanetworkopen.2025.44812 (PMC12658655; doi:10.1001/jamanetworkopen.2025.44812)
Supplement: Supplement 2. — Data Sharing Statement [file jamanetwopen-e2544812-s002.pdf]

Data Sharing Statement

Taylor. Risk of Late-Onset Depression in Long-Term Survivors of Breast, Prostate, and Colorectal Cancer. *JAMA Netw Open*. Published November 26, 2025.
doi:10.1001/jamanetworkopen.2025.44812

Data
Data available: No
